# A lack of peptide binding and decreased thermostability suggests that the CASKIN2 scaffolding protein SH3 domain may be vestigial

**DOI:** 10.1186/s12900-016-0065-5

**Published:** 2016-09-13

**Authors:** Jamie J. Kwan, Logan W. Donaldson

**Affiliations:** 1 Department of Biology, York University, 4700 Keele Street, Toronto, ON M3J 1P3 Canada; 2 Present Address: McEwen Centre for Regenerative Medicine, Ontario Cancer Institute, 101 College Street, Toronto, ON M5G 1L7 Canada

**Keywords:** SH3 domain, NMR spectroscopy, Molecular modeling

## Abstract

**Background:**

CASKIN2 is a neuronal signaling scaffolding protein comprised of multiple ankyrin repeats, two SAM domains, and one SH3 domain. The CASKIN2 SH3 domain for an NMR structural determination because its peptide-binding cleft appeared to deviate from the repertoire of aromatic enriched amino acids that typically bind polyproline-rich sequences.

**Results:**

The structure demonstrated that two non-canonical basic amino acids (K290/R319) in the binding cleft were accommodated well in the SH3 fold. An K290Y/R319W double mutant restoring the typical aromatic amino acids found in the binding cleft resulted in a 20 °C relative increase in the thermal stability. Considering the reduced stability, we speculated that the CASKIN2 SH3 could be a nonfunctional remnant in this scaffolding protein.

**Conclusions:**

While the NMR structure demonstrates that the CASKIN2 SH3 domain is folded, its cleft has suffered two substitutions that prevent it from binding typical polyproline ligands. This observation led us to additionally survey and describe other SH3 domains in the Protein Data Bank that may have similarly lost their ability to promote protein-protein interactions.

**Electronic supplementary material:**

The online version of this article (doi:10.1186/s12900-016-0065-5) contains supplementary material, which is available to authorized users.

## Background

**Fig. 1:**
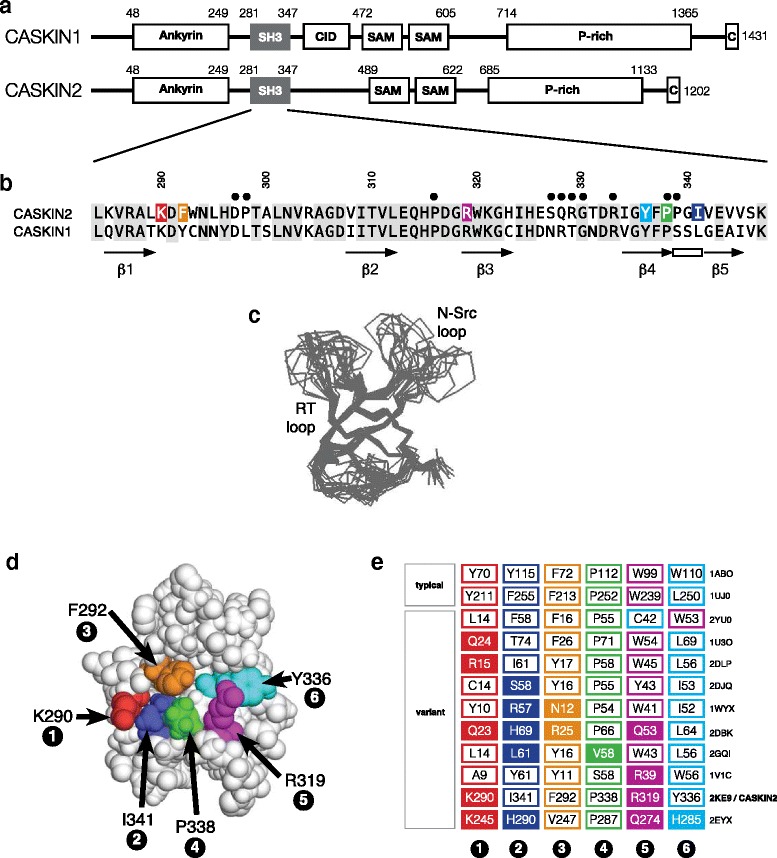
Domain organization of CASKIN2 and structure features of its SH3 domain. **a** CASKIN2, unlike CASKIN1, cannot bind the CASK scaffolding protein due to the absence of a CASK interacting domain (CID). The CASKIN sequences diverge in a proline rich (P-rich region), ultimately ending with a conserved C-terminal sequence of unknown significance. **b** Sequence comparison of the CASKIN2 and CASKIN1 SH3 domains. Black dots indicate amide resonances that could not be assigned, characteristic of intermediate (μs-ms) motions. Secondary structure of the CASKIN2 SH3 domain is shown below its sequence. Six amino acids constituting the canonical binding cleft are colored. **c** Cα superposition of the ensemble of lowest energy structures submitted to the Protein Data Bank (PDB: 2KE9) **d** Among the six amino acids in a typical peptide binding cleft, position 1 (K290) and position 5 (R319) are non-canonical. **e** A survey of binding clefts from SH3 domains in the Protein Data Bank that vary from the typical compliment of aliphatic and aromatic amino acids exemplified by Abl (PDB: 1ABO). From the survey, SH3 domains are observed to bear substitutions at one or more of the six positions (indicated by shading)

A pair of neuronal scaffolding proteins, represented in humans by CASKIN1 and CASKIN2, participate in signaling pathways involved in axon guidance and the creation of neuromuscular junctions. While mammals possess two CASKIN homologs, *Drosophila* only possesses one form (*Ckn*) that differs in domain composition and *C. elegans* has no homolog at all leading to the idea that the general role of CASKINs may be to provide more complex signaling outcomes in higher organisms by recruiting additional sets of protein partners [1]. Typical for many scaffolding proteins [2, 3], CASKIN1 and CASKIN2 are composed entirely of protein-protein interaction domains including six ankyrin repeats, an SH3 domain and tandem SAM domain (Fig. 1a). The C-terminal half of the protein, spanning over 600 residues and only partially conserved, is characterized by a proline-rich segment of unknown function [4].

CASKIN1, and CASKIN2 by homology, were named by their ability to serve as ligands of the MAGUK protein, CASK, a prominent signaling protein linked to calcium mediated signaling events, actin microfilament assembly, and synaptic communication through the neurexin cell surface receptor [4, 5]. Later structural studies of CASKIN1 revealed that the CASK interaction domain (CID), shared with X11/Mint and located in a region between the SH3 and SAM domains, was not present in CASKIN2. Thus, the namesake of CASKIN2 is misleading because it is unable to bind the calmodulin kinase domain of CASK [6]. This distinction lead us to speculate that despite their organizational similarity, CASKIN1 and CASKIN2 may have diverged with respect to their scaffolding functions in neurons [7].

Owing to the prominence of CASK in the development of neurological disease [8, 9], research to date has concentrated on CASKIN1 [10–12]. In this report, we have concentrated on the CASKIN2 SH3 domain to explore the question of whether functional differences between CASKIN1 and CASKIN2 extend to the structural level. Building upon the NMR solution structure of the CASKIN2 SH3 domain, we demonstrate that the typical binding cleft has two hydrophobic amino acid substitutions that do not affect the overall fold, but do affect thermostability and the ability to interact with peptides. These observations led us to consider that the CASKIN2 SH3 domain was non-functional. When hydrophobic amino acids were reintroduced, thermostability increased and high affinity binding was restored towards a peptide ligand of one of CASKIN2’s most homologous family members.

## Results and discussion

### Domain organization and sequence similarity

The CASKIN1 and CASKIN2 SH3 domains are located at the same position within each protein and are approximately 60 % similar (Fig. 1b). Following the SH3 domain, CASKIN1 and CASKIN2 diverge in sequence, the notable differences being the lack of a CASK interaction domain (CID) in CASKIN2, as well as the length and composition of a carboxy terminal proline-rich region. Thus, depending on the perspective, CASKIN1 and CASKIN2 are simultaneously similar and different, and therefore warrant some caution when making functional assumptions about one protein in the absence of knowledge from the other.

### Structure of the CASKIN2 SH3 domain

We began this study with a NMR solution structure determination of the CASKIN2 SH3 domain to provide a high-resolution framework for exploring how the SH3 domain may interact with ligands and react to post-translational modifications. Backbone atom precision for the ensemble of structures was 0.60 ± 0.14 Å, consistent with the number of experimental observations used in the structure calculation. Complete statistics pertaining to the experimental observations and structural quality are presented in Table 1. From the ensemble of the lowest energy structures, the RT and N-Src loops presented a high degree of disorder (Fig. 1c), presumably due to the absence of a ligand. Experimentally, the decrease in backbone atom precision is manifested by missing backbone assignments (D297, S327-D330, and R333) and short-range NOE observations (depicted as dots above the sequence in Fig. 1). A survey of the PDB for similar structures revealed the SH3 domains from human KIAA17833 and yeast NBP2 (both from structural proteomics studies) along with the well characterized SH3 domain from STAM2 [13] (Table 2).

**Table 1:** Restraints and statistics for the ensemble of 15 structures

NOE restraints		
	Total	413
	Intraresidue (|i – j| = 0)	204
	Sequential (|i – j| = 1)	102
	Medium range (1 < |i – j| < 5)	8
	Long range (|i – j| ≥ 5)	99
Additional restraints		
	Hydrogen bond distance restraints	32
	Backbone angle torsion angle restraints	74
RMS deviations^a^		
	Bonds	0.0123 ± 0.0003
	Angles	1.2827 ± 0.0363
	Improper angles	1.6905 ± 0.1112
	Dihedral angles	0.2015 ± 0.1411
RMS violations		
	NOE restraints > 0.5 Å	0.0 ± 0.0
	NOE restraints > 0.3 Å	2.3 ± 1.2
	NOE restraints > 0.1 Å	25.5 ± 4.1
	Dihedral angles > 5°	6.2 ± 0.6
Ramachandran analysis for ordered residues^b^		
	Most favored regions	95.2 %
	Additional allowed regions	4.8 %
	Generously allowed regions	0.0 %
	Disallowed regions	0.0 %

^a^As reported by XPLOR-NIH 2.30 using the standard protein force field

^b^As reported by PROCHECK for residues 284–292, 298–312, 320–324, 334–343

**Table 2:** Structurally similar proteins to the CASKIN2 SH3 domain

PDB	Protein	Source	RMSD	Aligned	Identity
2DLP	KIAA17833	NMR	1.1 Å	49 aa	29 %
1YN8	NBP2	X-ray	1.2 Å	49 aa	21 %
1UJ0	STAM2 + UBPY peptide	X-ray	1.6 Å	57 aa	33 %

### The CASKIN2 SH3 domain peptide binding cleft

A typical SH3 domain presents a binding cleft comprised of six, nearly linearly arranged, hydrophobic amino acids. As shown in Fig. 1d, the six amino acids in CASKIN2 are K290, I341, F292, P338, R319, and Y336. Notably, there is strong deviation at the first and fifth positions, denoted by K290 and R319. Since the side chains of the SH3 domain binding cleft are surface exposed, K290 and R319 do not affect the protein fold, and presumably, this is also the case for CASKIN1. Given that the binding cleft deviates at two positions from the typical set of amino acids observed in PxxP type SH3 domains like Abl kinase [14] and RxxK type SH3 domains like STAM2 [13], we hypothesized that the CASKIN2 SH3 domain might bind an unconventional peptide ligand. To answer this question, we performed four successive rounds of panning a 6xHis_−_-tagged CASKIN2 SH3 domain against a commercial bacteriophage 12-mer peptide display library (PHD-12; Novagen). A comparison of eight peptides from the last round of panning revealed a Pxx[L/M/W] motif in many of the candidates [presented in Additional file 1]. However, upon testing this motif with two chemically synthesized peptides and one reversed peptide as a control, we did not observe any binding in NMR based titrations suggesting that the library may have identified a very weak interaction. Since the phage display method failed to identify a ligand for the CASKIN2 SH3 domain, it led us to consider that the CASKIN2 SH3 domain might be a non-functional remnant of the protein.

Figure 1e presents a comparison of high resolution SH3 domain structures from the Protein Data Bank with an emphasis on representatives having peptide binding clefts that deviate from the usual complement of aliphatic and aromatic amino acids, and consequently, have no ligands reported for them. The Abl SH3 domain leads a set of sequences in Fig. 1e as a point of reference and prototype for the typical array of aromatic and aliphatic amino acids that populate the six positions of the binding cleft. Next to Abl in the presentation is the RUN/TBC1 domain containing protein-3 SH3 domain (PDB: 2YUO) that swaps an aromatic/aliphatic pair at positions 5 and 6 of the cleft with a compensatory aliphatic/aromatic pair. A survey of the remaining representatives in the figure demonstrate that it is possible for each of the six binding cleft positions to tolerate a hydrophilic or charged amino acid substitution. The Crk-II SH3(2) domain represents the most extreme case with substitutions at positions 1, 2, 5 and 6. Loss of ligand binding in the Crk-II SH3(2) domain in the context of the full adaptor protein is consistent with a stabilizing function rather than a regulatory function in this domain [15].

### Substitution mutagenesis of the CASKIN2 peptide binding cleft

**Fig. 2:**
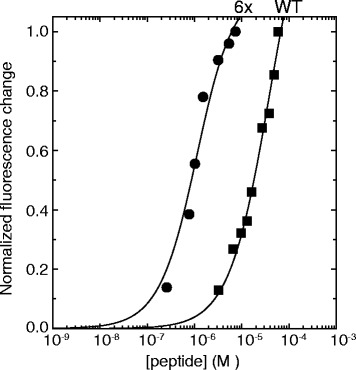
An SH3-6x mutant (K290Y, H296E, A300E, R319W, Y336L, I341F) mimics the binding cleft of the STAM2 SH3 domain in a CASKIN2 SH3 domain framework and consequently, high affinity towards a STAM2 ligand (YTPMVRENKPP) derived from the protein, UBPY. From direct curve fitting against a binding equation describing a 1:1 protein to peptide complex, the dissociation constants (*K*_*d*_) of wild type CASKIN2 SH3 domain and 6x mutant are 39 ± 6 μM and 220 ± 20 nM, respectively

A CASKIN2 SH3 domain double mutant (K290Y, R319W) that we termed SH3-2x, was expressed to reintroduce a full complement of aromatic amino acids in the peptide binding cleft. Using circular dichroism (CD) spectroscopy, the SH3-2x protein demonstrated a 20 °C increase in its thermal denaturation midpoint (Table 3). This observation suggests that aromatics in these two positions contribute to the stability of the SH3 fold. Given the high degree of structural similarity to the STAM2 SH3 domain in complex with a UBPY peptide [16], we introduced four more substitutions into the SH3-2x framework to produce a full mimic of the STAM2 interaction in the CASKIN SH3 domain. Overall, this new mutant termed SH3-6x, contains two substitutions to reintroduce aromatics (K290Y, R319W), two minor substitutions that maintain hydrophobicity (Y336L, I341F) and two substitutions in the RT-loop (H296E, A300E) that provide important complementary ionic contacts to the UBPY peptide ligand in STAM2. Since the SH3-2x mutant was a subset of the SH3-6x mutant, the expressed protein had a similar thermal denaturation midpoint of 70 °C (Table 3). When tested against a Class-III UBPY peptide containing an RxxKP motif, high affinity binding (0.46 ± 0.11 μM) was observed (Fig. 2) in the same order of magnitude as another Class-III SH3 domain GADS with an SLP-76 peptide ligand [17]. As expected, the wild type CASKIN2 SH3 domain had an over 100 fold less affinity for the UBPY peptide. While ultralow affinity SH3 domains with biological relevance have been reported, there is no evidence to suggest if CASKIN2 is among this group [18, 19].

**Table 3:** Thermal denaturation midpoints of CASKIN2 SH3 domain mutants

SH3	Mutation(s)	T_m_ (°C)
WT	none	50
2x	K290Y, R319W	70
6x	K290Y, H296E, A300E, R319W, Y336L, I341F	70

## Conclusions

The NMR structure of the CASKIN2 SH3 domain demonstrates a variant ligand binding cleft populated with charged amino acids. Based on this unique feature, we hypothesized that the CASKIN2 SH3 domain may bind a ligand that does not belong to any of the canonical, mainly proline-rich sequences that SH3 domains typically bind. Since a phage display failed to identify a ligand for the CASKIN2 SH3 domain, we concluded that it may indeed be non-functional. Increased thermal stability and ligand binding could be engineered back into the CASKIN2 SH3 domain sequence with relatively few amino acid substitutions. This results presented in the report emphasize the considerable versatility that SH3 domains, and signaling adaptor proteins, in general, have at their disposal.

## Methods

### Cloning, expression and protein purification

The human CASKIN2 SH3 domain (aa. 284–348; Uniprot Q8WXE0) was amplified by PCR from a human cDNA and inserted into the *BamHI* and *XhoI* restriction sites of pGEX4T2 (GE Life Sciences) followed by transformation into *E. coli* BL21:DE3 to produce a glutathione S-transferase (GST) tagged fusion protein with intervening thrombin protease cleavage site. Two CASKIN2 SH3 domain mutants, termed 2x and 6x, contained two aromatic substitutions to reconstitute a typical hydrophobic binding cleft and six substitutions mimicking the canonical binding cleft of the STAM2 SH3 domain. These mutants were made using Quikchange method (Agilent). All constructs were verified by sequencing at the York University Core Facility. Isotopic labeling of wild type GST-CASKIN2 SH3 domain and mutants for NMR spectroscopy was achieved by performing a 1.5 L fermentation in a minimal medium containing 1 g of ^15^NH_4_Cl as the sole nitrogen source and/or 3 g of ^13^C-glucose as the sole carbon source. The cell pellet was suspended in T300 buffer (20 mM Tris-HCl, 300 mM NaCl, 0.05 % NaN_3_) and lysed by French press. Purification of the GST-CASKIN2 SH3 protein was achieved by glutathione affinity chromatography (GE Biosciences). After an 8-h digestion with activated human thrombin (Sigma-Aldrich), the SH3 domain was resolved from the GST carrier protein by gel filtration chromatography (Sephacryl-100, HiLoad 16/60; GE Life Sciences). The final buffer for all analyses was phosphate buffered saline (PBS; 20 mM sodium phosphate, pH 7.8, 0.15 M NaCl, 0.05 % (w/v) sodium azide.

### Circular dichroism

Far UV spectra (190–260 nm) of CASKIN SH3 proteins at 20 μM in PBS were obtained on a Jasco J-810 spectropolarimeter and processed with Spectra Analysis 1.54.04 software. A midpoint thermal denaturation curve (T_m_) was generated by monitoring the ellipticity signal at 220 nm as the temperature was ramped from 20 to 90 °C at a rate of 1 °C/min.

### Peptide binding

A solution of peptide YTPMVRENKPP at > 90 % purity (Canpeptide; Montreal, QC) corresponding to the UBPY ligand in the STAM2 SH3 domain structure (PDB: 1UJ0) was titrated into 1 μM wild type CASKIN2 SH3 domain, a 6x CASKIN-SH3 domain mutant, or a reference cell containing buffer only (10 mM Tris-HCl, pH 7.5, 150 mM NaCl, 0.05 % sodium azide). Upon excitation at 280 nm, intrinsic fluorescence emission was measured at 340 nm corresponding to one partially buried tryptophan in the CASKIN2 SH3 domain. Each measurement was made three times and averaged.

### NMR spectroscopy and structure determination

All experiments were performed on a uniformly ^13^C,^15^N labeled sample of the CASKIN2 SH3 domain sample at 0.6 mM plus 10 % D_2_O. A conventional heteronuclear, triple-resonance strategy was employed with all experiments being acquired on an Agilent 600 MHz spectrometer equipped with a 5 mm cryoprobe. Backbone directed experiments : HNCACB and CBCAcoNH (^15^N_sw_ = 1450 Hz, ^15^N_pts_ = 28; ^13^C_sw_, = 11309 Hz, ^13^C_pts_ = 40), HNCO and HNcaCO (^15^N_sw_ = 1450 Hz, ^15^N_pts_ = 28; ^13^C_sw_, = 2262 Hz, ^13^C_pts_ = 28). Side chain directed experiments: HccoNH (^15^N_sw_ = 1450 Hz, ^15^N_pts_ = 28; ^1^H_sw_, = 6596 Hz, ^1^H_pts_ = 80), hCcoNH (^15^N_sw_ = 1450 Hz, ^15^N_pts_ = 28; ^13^C_sw_, = 11309 Hz, ^13^C_pts_ = 28), HCCH-TOCSY (^13^C_sw_, = 11309 Hz, ^13^C_pts_ = 28), aromatic HBcbcgCD and HBcbcgcdCE (^13^C_sw_, = 4524 Hz, ^13^C_pts_ = 32). Distance restrains were measures from peak volumes from a ^15^N-edited NOESY (^15^N_sw_ = 1450 Hz, ^15^N_pts_ = 28; ^1^H_sw_, = 6596 Hz, ^1^H_pts_ = 80, 100 ms mixing time) and ^13^C-edited aliphatic (^1^H_sw_, = 6596 Hz, ^1^H_pts_ = 80, ^13^C_sw_, = 2800 Hz, ^13^C_pts_ = 36, 100 ms mixing time) and aromatic 3D-NOESYs aliphatic (^1^H_sw_, = 6596 Hz, ^1^H_pts_ = 80, ^13^C_sw_, = 2800 Hz, ^13^C_pts_ = 24, 100 ms mixing time). Datasets were processed with NMRpipe [20] and interpreted with NMRView [21]. Distance restraints were obtained using CYANA. Backbone torsion angles were predicted from backbone chemical shift data with TALOS+ [22]. From an initial set of 500 structures calculated with CYANA 2.0 [23], the top 25 structures were selected with no NOE violations > 0.3 Å and no torsion angle violations < 5°. This ensemble was then subjected to additional refinement in explicit solvent [24] with XPLOR-NIH 2.30 [25] and 15 final lowest energy structures were selected for deposition to the Protein Data Bank.

## Additional file

Additional file 1: Application of phage display to identify a potential ligand for the CASKIN2 SH3 domain. (PDF 86 kb)

## Abbreviations

CASK: Calcium/calmodulin-dependent serine protein kinase
CASKIN2: CASK interacting protein-2
CD: Circular dichroism
CID: CASK interaction domain
GST: glutathione S-transferase
NMR: Nuclear magnetic resonance
PDB: Protein data bank
