# Supplementary material for: A lack of peptide binding and decreased thermostability suggests that the CASKIN2 scaffolding protein SH3 domain may be vestigial
Source: BMC Struct Biol. 2016 Sep 13;16(1):14. doi: 10.1186/s12900-016-0065-5 (PMC5020537; doi:10.1186/s12900-016-0065-5)
Supplement: Additional file 1: — Application of phage display to identify a potential ligand for the CASKIN2 SH3 domain. (PDF 86 kb) [file 12900_2016_65_MOESM1_ESM.pdf]

```
08      SPHHEHPPRPLI
06         WHXNPTTLPTXD
04       WHVPKIPLLMPT
11     HHFKLVNQPTPM
09        KHPMDPITWGWW
05      KPHHNNIPSVWS
01            HPQGWTWYSAPI
03           SSPGLWPAQRLATH

03*     SSHTALRQAPWLGP
```

Application of phage display to identify a potential ligand for the CASKIN2 SH3 domain. A preparation of 5 μM purfied 6xHis-tagged CASKIN2 SH3 domain was panned for four cycles against a PHD-12 library (Invitrogen) according to the instructions recommended by the manufacturer. M13 phage ssDNA was purified using a Qiaprep Spin M13 kit (Qiagen) and submitted for sequencing at the Hospital for Sick Children genomics facility (Toronto, ON). A selection of sequenced clones shared a weak consensus sequence of P-X-X-[LMW]. Peptides corresponding to #05 and #03, and the reverse sequence of the latter, 03*, were chemically synthesized by CanPeptide (Montreal, QC) and tested by titration into a ^{15}N-labeled preparation of wild type CASKIN2 SH3 domain. No binding was detected for any of the peptides suggesting that the either the interaction was very weak given the sensitivity of the NMR method.
